# *Failure to Cure* in Patients Undergoing Surgery for Gastric Cancer: A Nationwide Cohort Study

**DOI:** 10.1245/s10434-020-09510-6

**Published:** 2021-01-23

**Authors:** Daan M. Voeten, Leonie R. van der Werf, Janneke A. Wilschut, Linde A. D. Busweiler, Johanna W. van Sandick, Richard van Hillegersberg, Mark I. van Berge Henegouwen, on behalf of the Dutch Upper Gastrointestinal Cancer Audit Group

**Affiliations:** 1 Department of Surgery, Cancer Center Amsterdam, Amsterdam UMC, University of Amsterdam, Amsterdam, The Netherlands; 2 Scientific Bureau, Dutch Institute for Clinical Auditing, Leiden, The Netherlands; 3 Department of Surgery, Gelre Hospital, Apeldoorn, The Netherlands; 4 Department of Surgical Oncology, Antoni van Leeuwenhoek Hospital, Amsterdam, The Netherlands; 5 Department of Surgery, University Medical Center Utrecht, Utrecht, The Netherlands

## Abstract

**Background:**

This study aimed to describe the incidence of *failure to cure* (a composite outcome measure defined as surgery not meeting its initial aim), and the impact of hospital variation in the administration of neoadjuvant therapy on this outcome measure.

**Methods:**

All patients in the Dutch Upper Gastrointestinal Cancer Audit undergoing curatively intended gastric cancer surgery in 2011–2019 were included. *Failure to cure* was defined as (1) ‘open-close’ surgery; (2) irradical surgery (R1/R2); or (3) 30-day/in-hospital mortality. Case-mix-corrected funnel plots, based on multivariable logistic regression analyses, investigated hospital variation. The impact of a hospital’s tendency to administer neoadjuvant chemotherapy on the heterogeneity in *failure to cure* between hospitals was assessed based on median odds ratios and multilevel logistic regression analyses.

**Results:**

Some 3862 patients from 28 hospitals were included. *Failure to cure* was noted in 22.3% (hospital variation: 14.5–34.8%). After case-mix correction, two hospitals had significantly higher-than-expected *failure to cure* rates, and one hospital had a lower-than-expected rate. The *failure to cure* rate was significantly higher in hospitals with a low tendency to administer neoadjuvant chemotherapy. Approximately 29% of hospital variation in *failure to cure* could be attributed to different hospital policies regarding neoadjuvant therapy.

**Conclusions:**

*Failure to cure* has an incidence of 22% in patients undergoing gastric cancer surgery. Higher *failure to cure* rates were seen in centers administering less neoadjuvant chemotherapy, which confirms the Dutch guideline recommendation on the administration of neoadjuvant chemotherapy. *Failure to cure* provides short loop feedback and can be used as a quality indicator in surgical audits.

**Supplementary Information:**

The online version contains supplementary material available at 10.1245/s10434-020-09510-6.

Gastric cancer is the fifth most prevalent and third most lethal type of cancer worldwide.^1^ Surgical resection combined with perioperative chemotherapy is the cornerstone of potentially curative treatment. Five-year overall survival rates after such treatment vary at around 40%.^2^^–^4 However, curative surgical treatment is not always possible due to metastatic disease, local irresectability, or condition of the patient. In the Netherlands, there is an increasing awareness of a significant hospital variation in the selection of surgical candidates,^5^ and there also exists significant hospital variation in the administration of perioperative chemotherapy.^6^,^7^

*Failure to cure* is a composite outcome measure first defined by Clavien in 1992 as ‘surgery not meeting its initial aim’.^8^,^9^ It was recently added as a quality indicator to the Dutch Upper Gastrointestinal Cancer Audit (DUCA) based on a recent study describing *failure to cure* as an outcome measure capable of identifying significant hospital variation in the quality of esophageal cancer surgery.^10^ The *failure to cure* composite outcome measure might also be important in identifying hospital variation in other low-incidence surgical procedures such as oncologic gastrectomy.^11^ While most gastric cancer literature describes single outcome measures strictly focusing on surgical quality, the composite outcome measure *failure to cure* does not only reflect the quality of the surgical procedure itself but also evaluates preoperative diagnostics, the selection of patients eligible for surgery, and (multidisciplinary team/shared) decision making; however, as yet, *failure to cure* has not been described for gastric cancer patients. For patients undergoing gastric cancer surgery, *failure to cure* comprises either (1) futile surgery (‘open-close’) due to intraoperative distant metastasis or locally irresectable disease; (2) an histogical irradical resection; and/or (3) postoperative mortality.

The primary aim of this study was to describe the incidence of *failure to cure* in patients undergoing gastric cancer surgery, and to identify possible hospital variation, while the secondary aim was to investigate the impact of hospital policies towards the administration of neoadjuvant chemotherapy on *failure to cure.* The current study hypothesized that the outcome measure *failure to cure* is capable of identifying hospital variation in quality of care after gastric cancer surgery. In addition, it hypothesized that non-administration of neoadjuvant chemotherapy negatively influences *failure to cure* rates.

## Methods

### Study Design

In this retrospective nationwide cohort study, data from the DUCA were used. The DUCA is a nationwide mandatory audit wherein all patients with esophageal or gastric cancer undergoing surgery with the intent of resection are registered.^12^ The DUCA dataset has been verified; data completeness was estimated at 99.2%, and outcome measure accuracy ranged from 95.3 to 100%.^13^ For the current study, no ethical approval or informed consent was required under Dutch law. The DUCA Scientific Committee approved this study’s protocol, and the study was conducted in accordance with the Strengthening the Reporting of Observational Studies in Epidemiology (STROBE) guidelines.^14^

### Patient Selection

All patients who underwent gastric cancer surgery with curative intent between 1 January 2011 and 31 December 2019 were included. In the DUCA, gastroesophageal junction and cardia carcinomas are registered as esophageal cancer and were therefore excluded. To minimize statistical artefacts due to small sample sizes in hospital variation analysis, patients were excluded if they had undergone surgery in a hospital where fewer than 25 gastric cancer resections were performed throughout the entire study period. In case of missing data in components essential for the calculation of *failure to cure* (as described below), patients were also excluded.

### Definition of Failure to Cure

In accordance with previous literature describing *failure to cure* as an outcome measure for esophageal cancer surgery, the current study defined *failure to cure* as (1) futile surgery due to intraoperative distant metastasis or local tumor irresectability; (2) microscopically or macroscopically incomplete resection (R1/R2); or (3) 30-day and/or in-hospital mortality (i.e. mortality during the primary admission or, in case of discharge, until 30 days postoperatively).^10^ As each of these single outcome measures is measurable over a short period of time, *failure to cure* provides short-loop feedback that is essential for its use in a clinical audit. In addition, as all three single measures are also registered in the DUCA separately, clinicians have insight into the exact areas for improvement. However, combining all three measures into one composite measure enhances visibility of hospital variation for low-incident surgical procedures.

### Variables for Analyses

The following patient, tumor and treatment characteristics were used in the analyses: sex (male, female), age in years (< 65, 65–75, > 75), preoperative weight loss in kilograms (none, 1–5, 6–10, > 10), body mass index (< 20, 20–25, 26–30, > 30), American Society of Anesthesiologists (ASA) score (I, II, III+), Charlson Comorbidity Index^15^ (0, 1, 2+), previous esophageal, gastric, or hiatal surgery (no, yes), tumor location (corpus, fundus, antrum, pylorus, total stomach, rest stomach/anastomosis, unknown location), clinical T stage (T0–2, T3–4, Tx), clinical N stage (N0, N+, Nx), diagnostic laparoscopy (no, yes), endoscopic ultrasound (no, yes), neoadjuvant therapy (chemotherapy, none, other), surgical procedure (minimally invasive, open) and year of resection (before 2016, 2016 and later; this cut-off was used since the use of diagnostic laparoscopy increased significantly in 2016 in the Netherlands^16^ and because hospital volumes stabilized in 2016). After the Dutch volume threshold of 20 annual gastrectomies was introduced in 2011, centralization took place in the Netherlands. This resulted in a decrease in the number of gastrectomy centers from 34 in 2011 to 20 in 2017.^17^ Hospital volumes stabilized in 2016.^17^ Total annual gastrectomy hospital volume in the year of surgery was assigned to each patient and thereafter categorized into < 20, 20–40, > 40, and also used as variables for analyses.

### Statistical Analyses

The percentage of patients with *failure to cure* was described at both the national and hospital level. Depending on group sizes, the Chi square test or Fisher’s exact test was used to compare baseline characteristics between patients with and those without *failure to cure*. Univariable logistic regression analysis was used to identify patient, tumor, treatment, and hospital characteristics associated with *failure to cure*. All factors with a *p* value < 0.10 were added to a multilevel multivariable logistic regression model. The two-level model corrected for unmeasured hospital differences. Next, hospital variation corrected for baseline differences was investigated. The expected (E) number of patients with *failure to cure* was estimated for each hospital using multivariable logistic regression based on the patient and tumor characteristics described above. Thus, the expected number depended on the individual hospital’s case-mix. A case-mix-corrected funnel plot presented the observed (O) divided by the expected (E) number of *failures to cure* (O/E ratio) on the y-axis and the expected (E) number of *failures to cure* on the x-axis.^18^,^19^ An O/E ratio higher than 1 indicates a higher-than-expected *failure to cure* rate, whereas an O/E ratio below 1 indicates a lower-than-expected proportion. Ninety-five percent confidence intervals were computed around the benchmark (observed = expected).

### Impact of Neoadjuvant Therapy on Failure to Cure

As in the Dutch guideline neoadjuvant therapy is only recommended for patients with stage II disease or higher, all analyses described above were repeated for this cohort of patients (including stage X).^20^,^21^ A case-mix-corrected funnel plot showing each hospital’s tendency to administer neoadjuvant chemotherapy was contrived using the methods described above. The O/E ratio (continuous variable) was added as a fixed-effect variable to a multilevel multivariable logistic regression model (including the baseline patient and tumor characteristics associated with *failure to cure* from previous univariable regression analyses) to assess the association between *failure to cure* and the tendency to administer neoadjuvant chemotherapy. To check for linearity, the squared O/E ratio was added to the model and its performance was assessed using the likelihood ratio test.

The method described by Merlo et al. was used to quantify the proportion of hospital variation in *failure to cure* caused by differences in neoadjuvant chemotherapy policies.^22^ In short, median odds ratios (mOR) for *failure to cure* were calculated in three multilevel models. mOR can be interpreted as the odds when randomly moving to another hospital. Only patients eligible for neoadjuvant therapy were included for these analyses. The three models were:

1. An ‘empty’ model with *failure to cure* as the dependent variable, including only hospital ID as a random effect.
2. Patient and tumor characteristics were added to model (1).
3. The O/E ratio was added to model (2) to investigate the extent to which hospital variation in *failure to cure* was explained by differences in hospital policies towards administering neoadjuvant chemotherapy.

To objectify the proportion of hospital variation in *failure to cure* caused by the hospital variation in the administration of chemotherapy, the proportional change in variance (PCV) was calculated as shown in Eq. 1:^23^

1 $${\text{PCV}} = \frac{{{\text{variance}}\;{\text{model}}\;{\text{ii}} - {\text{variance}}\;{\text{model}}\;{\text{iii}}}}{{{\text{variance}}\;{\text{model}}\;{\text{ii}}}}$$

All *p*-values were based on two-sided tests, and a *p* value < 0.05 was considered statistically significant. Missing items were analyzed as separate variable options if ≥ 5%, and were excluded from multivariable analyses when < 5%. The presence of multicollinearity was assessed in all multivariable analyses by calculation of the variance inflation factor (VIF). Absence of multicollinearity was assumed when the VIF was ≤ 2.5. R-studio version 1.2.5019 was used to perform all statistical analyses (The R Foundation for Statistical Computing, Vienna, Austria.^24^

## Results

**Fig. 1:**
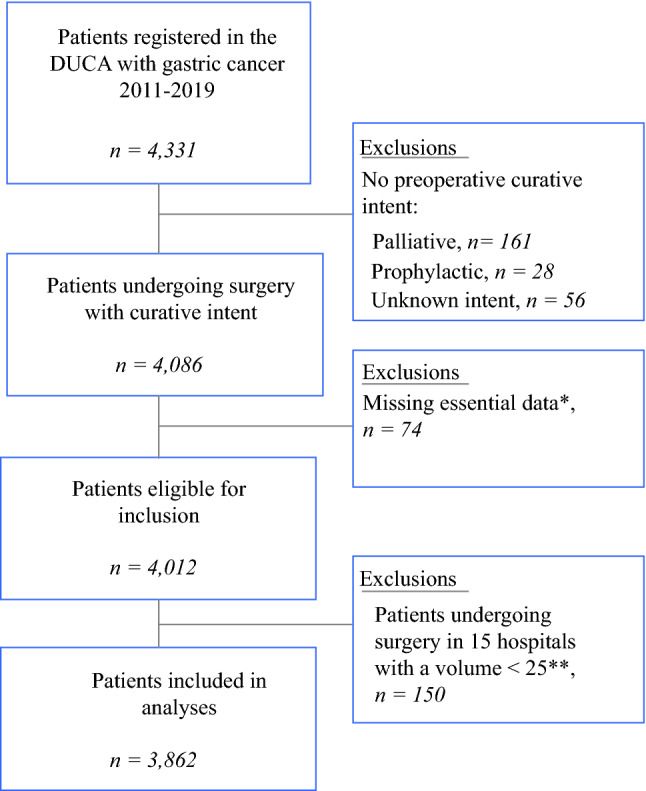
Study selection process. *Essential data: essential components for the calculation of *failure to cure* (pathological resection margin, nature of the surgery as defined by the surgeon at the end of the operation, and 30-day/in-hospital mortality). **Patients undergoing surgery in a hospital with a hospital volume of < 25 during the entire study period (2011–2019). *DUCA* Dutch Upper Gastrointestinal Cancer Audit

A total of 3862 gastric cancer patients met the inclusion criteria (Fig. 1). *Failure to cure* was noted in 861 (22.3%) patients. Surgery was futile in 326 patients (8.4%) due to intraoperative distant metastasis (141 patients), locally irresectable disease (81 patients), both local and distant irresectability (66 patients), intraoperative unstable condition of the patient (6 patients), or other/missing reasons (32 patients). In 347 patients (9.0%) the resection was irradical (R1: 276 patients; R2: 71 patients), and postoperative mortality occurred in 188 patients (4.9%).

### Factors Associated with Failure to Cure

Baseline patient, tumor, treatment, and hospital characteristics of patients with and those without *failure to cure* are depicted in Table 1. In multilevel multivariable logistic regression analyses, preoperative weight loss, total stomach tumor location, T3–4 or Tx, N+, and no neoadjuvant therapy were associated with *failure to cure* (Table 2).

**Table 1:** Patient, tumor, treatment and hospital characteristics of patients with and without failure to cure after gastric cancer surgery

	Total	Failure to cure		*p* Value (Chi square)
		Yes	No	
Total	3862	861 (22.3)	3001 (77.7)	
*Sex*				0.97
Male	2431 (62.9)	541 (62.8)	1890 (63.0)	
Female	1428 (37.0)	317 (36.8)	1111 (37.0)	
Missing	3 (0.1)	3 (0.3)	0 (0.0)	
*Age, years*				< 0.01
< 65	1164 (30.1)	227 (26.4)	937 (31.2)	
65–75	1424 (36.9)	308 (35.8)	1116 (37.2)	
> 75	1270 (32.9)	325 (37.7)	945 (31.5)	
Missing	4 (0.1)	1 (0.1)	3 (0.1)	
*Preoperative weight loss, kg*				< 0.01
None	1067 (27.6)	137 (15.9)	930 (31.0)	
1–5	936 (24.2)	199 (23.1)	737 (24.6)	
6–10	882 (22.8)	249 (28.9)	633 (21.1)	
> 10	454 (11.8)	161 (18.7)	293 (9.8)	
Missing	523 (13.5	115 (13.4)	408 (13.6)	
*Body mass index*				< 0.01
< 20	324 (8.4)	88 (10.2)	236 (7.9)	
20–25	2017 (52.2)	472 (54.8)	1545 (51.5)	
26–30	1059 (27.4)	214 (24.9)	845 (28.2)	
> 30	379 (9.8)	64 (7.4)	315 (10.5)	
Missing	83 (2.1)	23 (2.7)	60 (2.0)	
*ASA score*				< 0.01
I	467 (12.1)	87 (10.1)	380 (12.7)	
II	2115 (54.8)	449 (52.1)	1666 (55.5)	
III+	1258 (32.6)	319 (37.0)	939 (31.3)	
Missing	22 (0.6)	6 (0.7)	16 (0.5)	
*CCI*				< 0.01
0	1658 (42.9)	346 (40.2)	1312 (43.7)	
1	923 (23.9)	188 (21.8)	735 (24.5)	
2+	1280 (33.1)	327 (38.0)	953 (31.8)	
Missing	1 (0.0)	0 (0.0)	1 (0.0)	
*Previous esophageal or gastric surgery*				0.03
No	3553 (92.0)	777 (90.2)	2776 (92.5)	
Yes	287 (7.4)	79 (9.2)	208 (6.9)	
Unknown/missing	22 (0.6)	5 (0.6)	17 (0.6)	
*Tumor location*				< 0.01
Corpus	1204 (31.2)	238 (27.6)	966 (32.2)	
Fundus	354 (9.2)	72 (8.4)	282 (9.4)	
Antrum	1507 (39.0)	307 (35.7)	1200 (40.0)	
Pylorus	323 (8.4)	74 (8.6)	249 (8.3)	
Total stomach	215 (5.6)	101 (11.7)	114 (3.8)	
Rest stomach/anastomosis	161 (4.2)	50 (5.8)	111 (3.7)	
Unknown location	45 (1.2)	8 (0.9)	37 (1.2)	
Missing	53 (1.4)	11 (1.3)	42 (1.4)	
*Clinical tumor stage*^*a*^				< 0.01
T0–2	1045 (27.1)	117 (13.6)	928 (30.9)	
T3–4	1947 (50.4)	527 (61.2)	1420 (47.3)	
Tx	817 (21.2)	201 (23.3)	616 (20.5)	
Missing	53 (1.4)	16 (1.9)	37 (1.2)	
*Clinical node stage*^*a*^				< 0.01
N0	1928 (49.9)	352 (40.9)	1576 (52.5)	
N+	1482 (38.4)	394 (45.8)	1088 (36.3)	
Nx	400 (10.4)	97 (11.3)	303 (10.1)	
Missing	52 (1.3)	18 (2.1)	34 (1.1)	
*Diagnostic laparoscopy*				0.11
No	2755 (71.3)	597 (69.3)	2158 (71.9)	
Yes	1034 (26.8)	249 (28.9)	785 (26.2)	
Missing	73 (1.9)	15 (1.7)	58 (1.9)	
*Endoscopic ultrasound*				0.64
No	2807 (72.7)	621 (72.1)	2186 (72.8)	
Yes	963 (24.9)	220 (25.6)	743 (24.8)	
Missing	92 (2.4)	20 (2.3)	72 (2.4)	
*Neoadjuvant therapy*				< 0.01
Chemotherapy	2154 (55.8)	395 (45.9)	1759 (58.6)	
None	1614 (41.8)	448 (52.0)	1166 (38.9)	
Other neoadjuvant therapy	87 (2.3)	15 (1.7)	72 (2.4)	
Missing	7 (0.2)	3 (0.3)	4 (0.1)	
*Surgical procedure*				< 0.01
Minimally invasive	1817 (47.0)	362 (42.0)	1455 (48.5)	
Open	2044 (52.9)	498 (57.8)	1546 (51.5)	
Missing	1 (0.0)	1 (0.1)	0 (0.0)	
*Hospital volume (gastric resections per year)*				< 0.01
< 20	1103 (28.6)	278 (32.3)	825 (27.5)	
20–39	2242 (58.1)	485 (56.3)	1757 (58.5)	
≥ 40	517 (13.4)	98 (11.4)	419 (14.0)	
*Year of resection*				< 0.01
Prior to 2016	2063 (53.4)	501 (58.2)	1562 (52.0)	
2016 and later	1795 (46.5)	359 (41.7)	1436 (47.9)	
Missing	4 (0.1)	1 (0.1)	3 (0.1)	

*ASA* American Society of Anesthesiologists, *CCI* Charlson Comorbidity Index

^a^In conformity with the 7th edition of the TNM classification rules

**Table 2:** Univariable and multilevel multivariable logistic regression model, nested for factorized hospital identification number, to assess the association of patient, tumor, hospital, and treatment characteristics with curative surgery (no failure to cure) for gastric cancer

Factor	*N*	Univariable analysis			Multilevel multivariable analysis		
		OR	95% CI	*p* value	aOR	95% CI	*p* Value
*Sex*							
Male	2431	1					
Female	1428	1.00	0.86–1.17	0.97			
*Age, years*							
< 65	1164	1			1		
65–75	1424	0.88	0.72–1.06	0.18	0.96	0.77–1.20	0.74
> 75	1270	0.70	0.58–0.85	< 0.01	0.99	0.78–1.27	0.97
*Preoperative weight loss, kg*							
None	1067	1			1		
1–5	936	0.55	0.43–0.69	< 0.01	0.62	0.48–0.80	< 0.01
6–10	882	0.37	0.30–0.47	< 0.01	0.43	0.34–0.56	< 0.01
> 10	454	0.27	0.21–0.35	< 0.01	0.33	0.24–0.44	< 0.01
Missing	523	0.52	0.40–0.69	< 0.01	0.62	0.45–0.84	< 0.01
*Body mass index*							
< 20	324	1			1		
20–25	2017	1.22	0.93–1.59	0.14	1.06	0.79–1.41	0.71
26–30	1059	1.47	1.10–1.96	< 0.01	1.15	0.84–1.58	0.38
> 30	379	1.84	1.28–2.65	< 0.01	1.36	0.91–2.05	0.14
*ASA score*							
I	467	1			1		
II	2115	0.85	0.65–1.09	0.21	0.90	0.67–1.21	0.48
III+	1258	0.67	0.51–0.88	< 0.01	0.81	0.58–1.12	0.20
*CCI*							
0	1658	1			1		
1	923	1.03	0.85–1.26	0.76	1.11	0.89–1.39	0.36
2+	1280	0.77	0.65–0.91	< 0.01	0.87	0.70–1.07	0.18
*Previous esophageal or gastric surgery*							
No	3553	1			1		
Yes	287	0.74	0.56–0.97	0.03	0.94	0.61–1.46	0.79
*Tumor location*							
Corpus	1204	1			1		
Fundus	354	0.96	0.72–1.30	0.81	1.00	0.72–1.38	0.98
Antrum	1507	0.96	0.80–1.16	0.70	1.02	0.83–1.25	0.85
Pylorus	323	0.83	0.62–1.12	0.21	1.00	0.72–1.38	0.99
Total stomach	215	0.28	0.21–0.38	< 0.01	0.30	0.21–0.41	< 0.01
Rest stomach/anastomosis	161	0.55	0.38–0.79	< 0.01	0.70	0.40–1.21	0.20
Unknown location	45	1.14	0.55–2.66	0.74	1.32	0.52–3.35	0.56
*Clinical tumor stage*^*a*^							
T0–2	1045	1			1		
T3–4	1947	0.34	0.27–0.42	< 0.01	0.40	0.31–0.51	< 0.01
Tx	817	0.39	0.30–0.50	< 0.01	0.47	0.35–0.63	< 0.01
*Clinical node stage*^*a*^							
N0	1928	1			1		
N+	1482	0.62	0.52–0.73	< 0.01	0.68	0.57–0.82	< 0.01
Nx	400	0.70	0.54–0.90	< 0.01	0.82	0.59–1.13	0.22
*Diagnostic laparoscopy*							
No	2755	1					
Yes	1034	0.87	0.74–1.03	0.11			
*Endoscopic ultrasound*							
No	2807	1					
Yes	963	0.96	0.81–1.14	0.64			
*Neoadjuvant therapy*							
Chemotherapy	2154	1			1		
None	1614	0.58	0.50–0.68	< 0.01	0.49	0.40–0.60	< 0.01
Other neoadjuvant therapy	87	1.08	0.63–1.97	0.80	1.05	0.57–1.91	0.89
*Surgical procedure*							
Minimally invasive	1817	1			1		
Open	2044	0.77	0.66–0.90	< 0.01	0.92	0.76–1.12	0.40
*Hospital volume (gastric resections per year)*							
< 20	1103	1			1		
20–39	2242	1.22	1.03–1.44	0.02	1.11	0.89–1.40	0.36
≥ 40	517	1.44	1.12–1.87	< 0.01	1.27	0.92–1.75	0.15
*Year of resection*							
Prior to 2016	2063	1			1		
2016 and later	1795	1.28	1.10–1.50	< 0.01	1.19	0.98–1.45	0.08

*ASA* American Society of Anesthesiologists, *CCI* Charlson Comorbidity Index, *OR* odds ratio, *CI* confidence interval, *aOR* adjusted odds ratio

^a^In conformity with the 7th edition of the TNM classification rules

### Hospital Variation

**Fig. 2:**
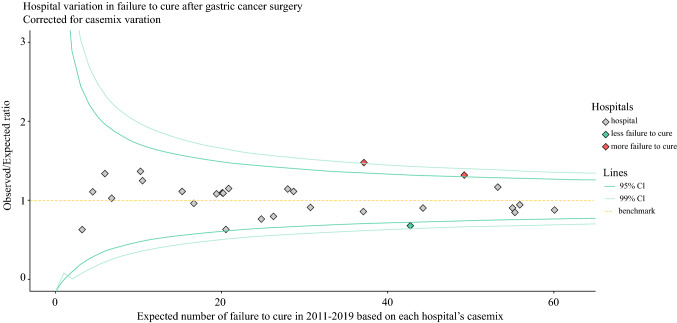
Case-mix-corrected funnel plot showing significant hospital variation in *failure to cure* after gastric cancer surgery. *CI* confidence interval

*Failure to cure* rates ranged from 14.5 to 34.8% among the 28 included hospitals. The case-mix-corrected hospital results are shown in Fig. 2. Two hospitals had significantly higher *failure to cure* rates than would be expected based on their case-mix. One hospital had a significantly lower-than-expected *failure to cure* percentage.

### Impact of Neoadjuvant Therapy on Failure to Cure

Of the 3862 included patients, a selection of 3034 (78.6%) patients from 26 hospitals had gastric cancer stage II or higher, of whom 770 (25.4%) had *failure to cure.* Baseline characteristics and multilevel multivariable logistic regression analyses are shown in electronic supplementary Tables 1 and 2. Also in this cohort of patients there was significant hospital variation in *failure to cure* (electronic supplementary Fig. 1).

**Fig. 3:**
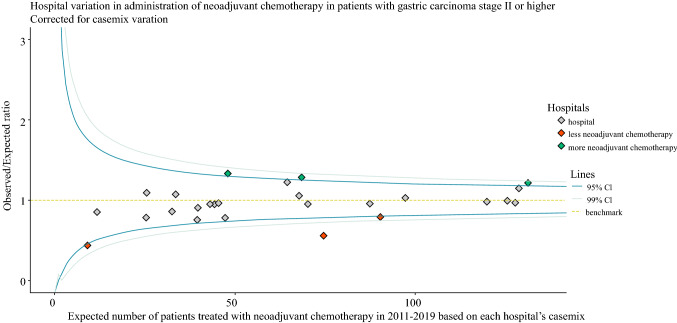
Case-mix-corrected funnel plot showing significant hospital variation in the administration of neoadjuvant chemotherapy for patients with stage II or higher gastric cancer. *CI* confidence interval

Figure 3 shows significant hospital variation in the administration of neoadjuvant chemotherapy after correction for case-mix. O/E ratios of the 26 hospitals ranged from 0.44 to 1.33, meaning that the percentage of patients undergoing surgery after having received neoadjuvant chemotherapy ranged from 26 to 79% among hospitals. *Failure to cure* was significantly associated with a low tendency to administer neoadjuvant chemotherapy after correction for patient- and tumor-related confounders and unmeasured hospital differences (odds ratio 2.01 for curative) [Table 3]. Adding the square of the O/E ratio did not lead to a better model fit (data not shown).

**Table 3:** Multivariable multilevel logistic regression analyses, nested for hospital identification number, to assess the association of each hospital’s tendency to administer neoadjuvant chemotherapy with failure to cure after surgery for gastric cancer stage II or higher, corrected for patient- and tumor-related confounders

Factor	Multivariable multilevel analysis with random effects for each hospital		
	aOR	95% CI	*p* Value
Observed/expected ratio of neoadjuvant chemotherapy administration when indicated for each hospital	2.01	1.02–3.94	0.04
*Age, years*			
< 65	1		
65–75	0.93	0.73–1.18	0.54
> 75	0.71	0.55–0.91	< 0.01
*Preoperative weight loss, kg*			
None	1		
1–5	0.68	0.51–0.91	0.01
6–10	0.47	0.35–0.62	< 0.01
> 10	0.33	0.24–0.45	< 0.01
Missing	0.63	0.44–0.89	< 0.01
*Body mass index*			
< 20	1		
20–25	1.03	0.74–1.43	0.86
26–30	1.05	0.74–1.50	0.77
> 30	1.28	0.81–2.02	0.29
*ASA score*			
I	1		
II	0.82	0.59–1.14	0.24
III+	0.70	0.49–1.00	0.05
*CCI*			
0	1		
1	1.11	0.87–1.42	0.40
2+	0.84	0.66–1.07	0.16
*Previous esophageal or gastric surgery*			
No	1		
Yes	0.87	0.54–1.39	0.56
*Tumor location*			
Corpus	1		
Fundus	1.06	0.74–1.53	0.74
Antrum	0.95	0.75–1.20	0.68
Pylorus	0.83	0.58–1.19	0.32
Total stomach	0.29	0.20–0.42	< 0.01
Rest stomach/anastomosis	0.75	0.41–1.38	0.36
Unknown location	1.09	0.42–2.84	0.86
*Clinical tumor stage*^*a*^			
T0–2	1		
T3–4	0.37	0.24–0.56	< 0.01
Tx	0.39	0.24–0.62	< 0.01
*Clinical node stage*^*a*^			
N0	1		
N+	0.66	0.53–0.82	< 0.01
Nx	0.84	0.59–1.18	0.31
*Year of resection*			
Prior to 2016	1		
2016 and later	1.28	1.05–1.56	0.01

*ASA* American Society of Anesthesiologists, *CCI* Charlson Comorbidity Index, *aOR* adjusted odds ratio, *CI* confidence interval

^a^In conformity with the 7th edition of the TNM classification rules

The mOR quantifying the differences between hospitals with respect to *failure to cure* are shown in Table 4. The PCV ($\frac{0.05856 - 0.04136}{0.05856}$) indicates that 29.4% of hospital differences in *failure to cure* can be explained by a hospitals’ tendency to administer neoadjuvant chemotherapy.

**Table 4:** Multilevel models performed to quantify the impact of differences in hospital neoadjuvant administration policy on failure to cure after gastric cancer surgery

	Model build-up	Variance	Median odds ratio
1.	Failure to cure ~ random effect of hospital	0.04901	1.235
2.	Failure to cure ~ patient and tumor characteristics + random effect of hospital	0.05856	1.260
3.	Failure to cure ~ hospital’s tendency to administer neoadjuvant chemotherapy + patient and tumor characteristics + random effect of hospital	0.04136	1.214

## Discussion

This study described the results of *failure to cure* in gastric cancer surgery. *Failure to cure* was noted in almost one of four patients who underwent potentially curative gastric cancer surgery in the Netherlands between 2011 and 2019. This ranged from 15 to 35% among the 28 Dutch hospitals performing gastric cancer surgery. After correction for case-mix, two hospitals had higher-than-expected *failure to cure* rates and one hospital had a significantly lower-than-expected rate. Separate analyses showed significant hospital variation in the use of neoadjuvant chemotherapy. *Failure to cure* significantly correlated with the administration of neoadjuvant chemotherapy, with lower *failure to cure* rates in hospitals where neoadjuvant chemotherapy was administered relatively often. In this study, it was estimated that about 29% of hospital variation in *failure to cure* was attributable to differences in neoadjuvant chemotherapy administration policies.

Composite outcome measures are easier to interpret for patients and have statistical advantages for low-incidence surgical procedures.^11^,^25^,^26^ Various composite outcome measures, such as textbook outcome, have already been described for gastrectomy patients.^27^ Even though the individual parameters of *failure to cure* have been described extensively, the composite outcome measure *failure to cure* has not been previously described for gastric cancer. It may be interpreted as unsuccessful surgery and does not only reflect surgical quality but also the preoperative processes in terms of both the quality of the combined diagnostic modalities and the selection of surgical candidates. Since several sub-items are combined, a composite outcome measure helps to discriminate between hospitals, especially in low-incidence surgery. As *failure to cure* is not composed of long-term surgical outcomes (e.g. survival), it can be measured over a short period and therefore provides short-loop feedback. This is essential for its use in clinical auditing. Currently, *failure to cure* is an internal quality indicator in the DUCA.^10^

One disadvantage of composite outcome measures is that they do not provide information on the individual parameters that could be improved to achieve better results. In addition, composite outcome measures do not take the unequal severity of its components into account (e.g. mortality is not considered worse than irradical surgery). Therefore, they should be used in addition to, but not replace, individual performance indicators. When outlier hospitals in *failure to cure* are identified, clinicians should consult the individual outcome measures for potential areas of improvement. In using *failure to cure* as a quality indicator, it is essential not to set the benchmark at 0% as this would lead to potentially harmful risk-averse behavior. In addition, in interpreting *failure to cure*, it is essential to understand that not having *failure to cure* is no assurance that cure has been achieved.

While the complication registration as proposed by Clavien in 1992 gained general acceptance, *failure to cure* was not widely accepted as an outcome measure. Most oncologic surgical literature focuses on the quality of the surgical procedure (in curatively treated patients) and studies often exclude open-close surgery or R2 resections. However, *failure to cure* not only focuses on operative quality but also on the quality of preoperative care. Therefore, revival of this outcome measure in comparing surgical quality in national audits is justified.

Numerous factors may explain hospital variation in *failure to cure* rates (15–35%). In the current study, a significant part of the hospital variation (29%) could be attributed to differences in hospital policies regarding neoadjuvant therapy. Neoadjuvant therapy might play a role in reducing *failure to cure*: irradical resection and futile surgery/open-close rates are lowered through downsizing the primary tumor or distant metastasis. In addition, radicality rates are obviously higher in complete responders to systemic neoadjuvant therapy. As the Dutch guideline advocates the use of neoadjuvant chemotherapy, it may only be omitted in frail patients. Therefore, residual confounding due to imperfect case-mix modeling might influence mortality, and therewith *failure to cure* rates. However, even though neoadjvuant chemotherapy is advocated in the guideline, two previous studies showed significant hospital variation in the administration of perioperative chemotherapy in the Netherlands, even after correction for patient- and tumor-related factors.^6^,^7^ Organizational and process factors also played a role in the administration of neoadjuvant therapy, and were not solely determined by patient-related characteristics. The study by Beck et al. suggested that expert centers more frequently administer neoadjuvant chemotherapy.^7^ The current study showed that prospects for successful surgery are lower in hospitals with a low tendency of administering neoadjuvant chemotherapy, even after statistical correction for patient- or tumor-related factors that might influence both neoadjuvant therapy administration rates and *failure to cure*. This confirms the Dutch guideline recommendation on the administration of neoadjuvant chemotherapy and suggests clinicians should be cautious in denying patients neoadjuvant chemotherapy. However, the hospital variation in the administration of neoadjuvant therapy indicates that some clinicians are more reluctant to administer neoadjuvant therapy than others. Combining the results of the current study and that by Beck et al., one could argue that referring patients to expert centers might be beneficial and that the administration of neoadjuvant therapy is a proxy for the overall quality of multimodal care provided by a hospital. Reduction of hospital variation in the administration of neoadjuvant chemotherapy might lead to a reduction in *failure to cure* rates. On the other hand, 71% of the hospital variation is attributable to other hospital differences, such as selection of surgical candidates. The proportion of patients with potentially curable gastric cancer undergoing surgery ranges from 57 to 78% among Dutch hospitals.^5^ Regional multidisciplinary team meetings, including multiple upper gastrointestinal specialists from different hospitals and specialties, may lead to greater uniformity in diagnostic work-up and selection of surgical and multimodal therapy candidates. Dutch upper gastrointestinal surgeons hold yearly ‘best practice’ meetings. Given the large hospital variation found in the current study, discussing preoperative work-up, decision making, and other clinical practices might induce nationwide improvement in *failure to cure* rates.^28^

Since 2016, the Dutch guideline encourages diagnostic laparoscopy in T3–T4 patients. Several studies demonstrated a positive effect of diagnostic laparoscopy on the prevention of futile surgery for gastric cancer;^29^,^30^ however, a recent Dutch study showed that open-close surgery rates are around 16% after performing a staging laparoscopy, indicating that distant metastasis develops between staging and potentially curative surgery (i.e. in the neoadjuvant therapy interval).^31^ The current study could not verify the role of diagnostic laparoscopy, as the DUCA only registers patients undergoing potentially curative surgery. Patients in whom diagnostic laparoscopy reveals metastasis and in whom curative surgery is waived are not registered. The outcomes of a Dutch prospective study regarding the value of diagnostic laparoscopy are awaited.^32^

The current study provides an overview of the results from the Dutch public healthcare system, which is (partially) centralized. External validity of these results in countries with private healthcare systems and/or non-centralized care is questionable. However, even in these different types of healthcare systems, *failure to cure* might be a powerful tool in the comparison of hospital performances. Especially in non-centralized countries where the incidence of the individual outcome measures in each hospital is low, combining outcomes into a composite measure has important statistical advantages.

This study showed a short-term mortality rate of 4.9% after gastric cancer surgery, which might be considered as high. Previous studies confirmed that post-gastrectomy mortality rates are relatively high in the Netherlands compared with other European countries,^33^,^34^ which may be a result of the relatively high tumor stages of patients undergoing surgery in the Netherlands. Mortality rates did improve in recent years, which might be a result of the centralization that occurred in parallel.^16^ Future research should focus on identifying reasons for postoperative mortality and ultimately establishing potential areas for improvement.

The present study has several limitations. First, this cohort study covers an 8-year inclusion period in which clinical practices have changed. In 2016, diagnostic laparoscopy rates rose significantly in the Netherlands, which limited the comparability of the cohorts before and after 2016. Therefore, we decided to add this variable to the multivariable models. The exact role of staging laparoscopy or endoscopic ultrasound could not be verified in the current study as only patients undergoing curative surgery after these diagnostic modalities are included in the DUCA registry. For defining their true value in preventing *failure to cure,* patients in whom curative surgery is waived based on these diagnostics should also be taken into consideration. Since the DUCA does not register restaging, the accuracy of primary staging and the impact of tumor remission status on *failure to cure* could not be investigated. The DUCA does not register tumor recurrence, which might also be considered a failure to cure. On the other hand, regarding its use in a surgical audit, it is essential that the outcome measure *failure to cure* can be measured over a short time period and that it provides short-loop feedback.

## Conclusion

In this nationwide cohort study, the composite outcome measure *failure to cure* was investigated for the first time for gastric cancer surgery. Next to the quality of surgery, it reflects the quality of the diagnostic work-up and the selection of patients eligible for surgery. *Failure to cure* was noted in 22.3% of gastric cancer patients who were operated with curative intent, and ranged from 15 to 35% among hospitals. Higher *failure to cure* rates were seen in centers administering less neoadjuvant chemotherapy, which confirms the Dutch guideline recommendation on the administration of neoadjuvant chemotherapy. This study warrants caution in denying patients neoadjuvant chemotherapy. Since *failure to cure* provides short-loop feedback, it can be used as a quality indicator in surgical audits.

## Supplementary Information

Supplementary material 1 (DOCX 98 kb)
