# Supplementary material for: Failure to Cure in Patients Undergoing Surgery for Gastric Cancer: A Nationwide Cohort Study
Source: Ann Surg Oncol. 2021 Jan 23;28(8):4484–96. doi: 10.1245/s10434-020-09510-6 (PMC8253712; doi:10.1245/s10434-020-09510-6)
Supplement: Supplementary file 1 — Supplementary material 1 (DOCX 98 kb) [file 10434_2020_9510_MOESM1_ESM.docx]

| *Online Supplements Table 1. Patient, tumor, treatment, and hospital characteristics of patients with and without failure to cure after surgery for gastric cancer stage II or higher.* |  | Total | Failure to cure |  | p-value  (χ^2^) |
| --- | --- | --- | --- | --- | --- |
|  |  |  | Yes | No |  |
|  |  | n (%) | n (%) | n (%) |  |
| Total |  | 3034 | 770 (25.4%) | 2264 (74.6%) |  |
| Sex |  |  |  |  | 0.89 |
|  | Male | 1917 (63.2%) | 484 (62.9%) | 1433 (63.3%) |  |
|  | Female | 1115 (36.8%) | 284 (36.9%) | 831 (36.7%) |  |
|  | Missing | 2 (0.1%) | 2 (0.3%) | 0 (0.0%) |  |
| Age in years |  |  |  |  | <0.01 |
|  | < 65 | 910 (30.0%) | 208 (27.0%) | 702 (31.0%) |  |
|  | 65-75 | 1120 (36.9%) | 264 (34.3%) | 856 (37.8%) |  |
|  | > 75 | 1001 (33.0%) | 297 (38.6%) | 704 (31.1%) |  |
|  | Missing | 3 (0.1%) | 1 (0.1%) | 2 (0.1%) |  |
| Preoperative weight loss in kg |  |  |  |  | <0.01 |
|  | None | 740 (24.4%) | 118 (15.3%) | 622 (27.5%) |  |
|  | 1-5 | 740 (24.4%) | 177 (23.0%) | 563 (24.9%) |  |
|  | 6-10 | 763 (25.1%) | 233 (30.3%) | 530 (23.4%) |  |
|  | >10 | 398 (13.1%) | 149 (19.4%) | 249 (11.0%) |  |
|  | Missing | 393 (13.0%) | 93 (12.1%) | 300 (13.3%) |  |
| Body Mass Index (BMI) |  |  |  |  | 0.04 |
|  | < 20 | 278 (9.2%) | 79 (10.3%) | 199 (8.8%) |  |
|  | 20-25 | 1608 (53.0%) | 425 (55.2%) | 1183 (52.3%) |  |
|  | 26-30 | 810 (26.7%) | 194 (25.2%) | 616 (27.2%) |  |
|  | > 30 | 285 (9.4%) | 56 (7.3%) | 229 (10.1%) |  |
|  | Missing | 53 (1.7%) | 16 (2.1%) | 37 (1.6%) |  |
| ASA score^A^ |  |  |  |  | 0.03 |
|  | I | 356 (11.7%) | 77 (10.0%) | 279 (12.3%) |  |
|  | II | 1672 (55.1%) | 411 (53.4%) | 1261 (55.7%) |  |
|  | III+ | 986 (32.5%) | 277 (36.0%) | 709 (31.3%) |  |
|  | Missing | 20 (0.7%) | 5 (0.6%) | 15 (0.7%) |  |
| CCI^B^ |  |  |  |  | 0.01 |
|  | 0 | 1317 (43.4%) | 315 (40.9%) | 1002 (44.3%) |  |
|  | 1 | 736 (24.3%) | 173 (22.5%) | 563 (24.9%) |  |
|  | 2+ | 981 (32.3%) | 282 (36.6%) | 699 (30.9%) |  |
| Previous esophageal or gastric surgery |  |  |  |  | 0.03 |
|  | No | 2791 (92.0%) | 696 (90.4%) | 2095 (92.5%) |  |
|  | Yes | 227 (7.5%) | 71 (9.2%) | 156 (6.9%) |  |
|  | Unknown/Missing | 16 (0.5%) | 3 (0.4%) | 13 (0.6%) |  |
| Tumor location |  |  |  |  | <0.01 |
|  | Corpus | 963 (31.7%) | 211 (27.4%) | 752 (33.2%) |  |
|  | Fundus | 304 (10.0%) | 65 (8.4%) | 239 (10.6%) |  |
|  | Antrum | 1110 (36.6%) | 273 (35.5%) | 837 (37.0%) |  |
|  | Pylorus | 251 (8.3%) | 67 (8.7%) | 184 (8.1%) |  |
|  | Total stomach | 190 (6.3%) | 96 (12.5%) | 94 (4.2%) |  |
|  | Rest stomach/anastomosis | 132 (4.4%) | 44 (5.7%) | 88 (3.9%) |  |
|  | Unknown location | 40 (1.3%) | 7 (0.9%) | 33 (1.5%) |  |
|  | Missing | 44 (1.5%) | 7 (0.9%) | 37 (1.6%) |  |
| Clinical Tumor stage^C^ |  |  |  |  | <0.01 |
|  | T0-2 | 255 (8.4%) | 35 (4.5%) | 220 (9.7%) |  |
|  | T3-4 | 1934 (63.7%) | 526 (68.3%) | 1408 (62.2%) |  |
|  | Tx | 792 (26.1%) | 193 (25.1%) | 599 (26.5%) |  |
|  | Missing | 53 (1.7%) | 16 (2.1%) | 37 (1.6%) |  |
| Clinical Node stage^C^ |  |  |  |  | 0.10 |
|  | N0 | 1148 (37.8%) | 268 (34.8%) | 880 (38.9%) |  |
|  | N+ | 1452 (47.9%) | 391 (50.8%) | 1061 (46.9%) |  |
|  | Nx | 383 (12.6%) | 93 (12.1%) | 290 (12.8%) |  |
|  | Missing | 51 (1.7%) | 18 (2.3%) | 33 (1.5%) |  |
| Diagnostic laparoscopy |  |  |  |  | 0.98 |
|  | No | 2064 (68.0%) | 524 (68.1%) | 1540 (68.0%) |  |
|  | Yes | 916 (30.2%) | 233 (30.3%) | 683 (30.2%) |  |
|  | Missing | 54 (1.8%) | 13 (1.7%) | 41 (1.8%) |  |
| Endoscopic ultrasound |  |  |  |  | 0.64 |
|  | No | 2217 (73.1%) | 558 (72.5%) | 1659 (73.3%) |  |
|  | Yes | 741 (24.4%) | 193 (25.1%) | 548 (24.2%) |  |
|  | Missing | 76 (2.5%) | 19 (2.5%) | 57 (2.5%) |  |
| Neoadjuvant therapy |  |  |  |  | <0.01 |
|  | Chemotherapy | 1785 (58.8%) | 361 (46.9%) | 1424 (62.9%) |  |
|  | None | 1172 (38.6%) | 395 (51.3%) | 777 (34.3%) |  |
|  | Other neoadjuvant therapy | 74 (2.4%) | 13 (1.7%) | 61 (2.7%) |  |
|  | Missing | 3 (0.1%) | 1 (0.1%) | 2 (0.1%) |  |
| Surgical procedure |  |  |  |  | <0.01 |
|  | Minimally invasive | 1431 (47.2%) | 327 (42.5%) | 1104 (48.8%) |  |
|  | Open | 1602 (52.8%) | 442 (57.4%) | 1160 (51.2%) |  |
|  | Missing | 1 (0.0%) | 1 (0.1%) | 0 (0.0%) |  |
| Hospital volume (gastric resections per year) |  |  |  |  | 0.04 |
|  | < 20 | 856 (28.2%) | 240 (31.2%) | 616 (27.2%) |  |
|  | 20-39 | 1786 (58.9%) | 445 (57.8%) | 1341 (59.2%) |  |
|  | ≥ 40 | 392 (12.9%) | 85 (11.0%) | 307 (13.6%) |  |
| Year of resection |  |  |  |  | <0.01 |
|  | < 2016 | 1626 (53.6%) | 445 (57.8%) | 1181 (52.2%) |  |
|  | 2016 and later | 1406 (46.3%) | 324 (42.1%) | 1082 (47.8%) |  |
|  | Missing | 2 (0.1%) | 1 (0.1%) | 1 (0.0%) |  |

**Legend:**

A American Society of Anesthesiologists Score

B Charlson Comorbidity Index

C In conformity with the 7th edition of the TNM rules for classification

|  |  |  | Univariable analysis | | | Multilevel multivariable analysis | | | | | | |
| --- | --- | --- | --- | --- | --- | --- | --- | --- | --- | --- | --- | --- |
| Factor |  | N | OR | CI (95%) | p-value | aOR^D^ | | | | CI (95%) | | p-value |
| Sex |  |  |  | | | |  |  |  | |  |  |
|  | Male | 1917 | 1 |  |  |  | | | |  | |  |
|  | Female | 1115 | 0.99 | 0.83 – 1.17 | 0.89 |  | | | |  | |  |
| Age in years |  |  |  |  |  |  | | | |  | |  |
|  | < 65 | 910 | 1 |  |  | 1 | | | |  | |  |
|  | 65- 75 | 1120 | 0.96 | 0.78 – 1.18 | 0.70 | 1.07 | | | | 0.85 – 1.36 | | 0.57 |
|  | > 75 | 1001 | 0.70 | 0.57 – 0.86 | <0.01 | 1.05 | | | | 0.81 – 1.37 | | 0.72 |
| Preoperative weight loss in kg |  |  |  |  |  |  | | | |  | |  |
|  | None | 740 | 1 |  |  | 1 | | | |  | |  |
|  | 1-5 | 740 | 0.60 | 0.46 – 0.78 | <0.01 | 0.65 | | | | 0.49 – 0.86 | | <0.01 |
|  | 6-10 | 763 | 0.43 | 0.34 – 0.55 | <0.01 | 0.45 | | | | 0.34 – 0.59 | | <0.01 |
|  | >10 | 398 | 0.32 | 0.24 – 0.42 | <0.01 | 0.35 | | | | 0.25 – 0.47 | | <0.01 |
|  | Missing | 393 | 0.61 | 0.45 – 0.83 | <0.01 | 0.63 | | | | 0.45 – 0.88 | | < 0.01 |
| Body Mass Index (BMI) |  |  |  |  |  |  | | | |  | |  |
|  | < 20 | 278 | 1 |  |  | 1 | | | |  | |  |
|  | 20-25 | 1608 | 1.11 | 0.83 – 1.46 | 0.49 | 0.98 | | | | 0.72 – 1.34 | | 0.90 |
|  | 26-30 | 810 | 1.26 | 0.92 – 1.71 | 0.14 | 1.02 | | | | 0.73 – 1.43 | | 0.90 |
|  | > 30 | 285 | 1.62 | 1.10 – 2.41 | 0.02 | 1.26 | | | | 0.82 – 1.96 | | 0.30 |
| ASA score^A^ |  |  |  |  |  |  | | | |  | |  |
|  | I | 356 | 1 |  |  | 1 | | | |  | |  |
|  | II | 1672 | 0.85 | 0.64 – 1.11 | 0.24 | 0.86 | | | | 0.63 – 1.18 | | 0.36 |
|  | III+ | 986 | 0.71 | 0.53 – 0.94 | 0.02 | 0.82 | | | | 0.58 – 1.17 | | 0.28 |
| CCI^B^ |  |  |  |  |  |  | | | |  | |  |
|  | 0 | 1317 | 1 |  |  | 1 | | | |  | |  |
|  | 1 | 736 | 1.02 | 0.83 – 1.27 | 0.83 | 1.11 | | | | 0.87 – 1.41 | | 0.40 |
|  | 2 + | 981 | 0.78 | 0.65 – 0.94 | <0.01 | 0.89 | | | | 0.71 – 1.11 | | 0.30 |
| Previous esophageal or gastric surgery |  |  |  |  |  |  | | | |  | |  |
|  | No | 2791 | 1 |  |  | 1 | | | |  | |  |
|  | Yes | 227 | 0.73 | 0.55 – 0.98 | 0.04 | 0.87 | | | | 0.55 – 1.36 | | 0.54 |
| Tumor location |  |  |  |  |  |  | | | |  | |  |
|  | Corpus | 963 | 1 |  |  | 1 | | | |  | |  |
|  | Fundus | 304 | 1.03 | 0.76 – 1.42 | 0.92 | 1.04 | | | | 0.74 – 1.46 | | 0.84 |
|  | Antrum | 1110 | 0.86 | 0.70 – 1.06 | 0.18 | 0.99 | | | | 0.79 – 1.24 | | 0.93 |
|  | Pylorus | 251 | 0.77 | 0.56 – 1.06 | 0.11 | 0.97 | | | | 0.68 – 1.37 | | 0.86 |
|  | Total stomach | 190 | 0.27 | 0.20 – 0.38 | <0.01 | 0.28 | | | | 0.20 – 0.40 | | <0.01 |
|  | Rest stomach/anastomosis | 132 | 0.56 | 0.38 – 0.84 | <0.01 | 0.77 | | | | 0.43 – 1.39 | | 0.39 |
|  | Unknown location | 40 | 1.32 | 0.61 – 3.30 | 0.66 | 1.12 | | | | 0.43 – 2.92 | | 0.81 |
| Clinical Tumor stage^C^ |  |  |  |  |  |  | | | |  | |  |
|  | T0-2 | 255 | 1 |  |  | 1 | | | |  | |  |
|  | T3-4 | 1934 | 0.43 | 0.29 – 0.61 | <0.01 | 0.40 | | | | 0.27 – 0.61 | | <0.01 |
|  | Tx | 792 | 0.49 | 0.33 – 0.72 | <0.01 | 0.49 | | | | 0.31 – 0.77 | | <0.01 |
| Clinical Node stage^C^ |  |  |  |  |  |  | | | |  | |  |
|  | N0 | 1148 | 1 |  |  | 1 | | | |  | |  |
|  | N+ | 1452 | 0.83 | 0.69– 0.99 | 0.04 | 0.67 | | | | 0.55 – 0.83 | | <0.01 |
|  | Nx | 383 | 0.95 | 0.73 – 1.25 | 0.71 | 0.83 | | | | 0.59 – 1.15 | | 0.26 |
| Diagnostic laparoscopy |  |  |  |  |  |  | | | |  | |  |
|  | No | 2064 | 1 |  |  |  | | | |  | |  |
|  | Yes | 916 | 1.00 | 0.83 – 1.19 | 0.98 |  | | | |  | |  |
| Endoscopic ultrasound |  |  |  |  |  |  | | | |  | |  |
|  | No | 2217 | 1 |  |  |  | | | |  | |  |
|  | Yes | 741 | 0.96 | 0.79 – 1.16 | 0.64 |  | | | |  | |  |
| Neoadjuvant therapy |  |  |  |  |  |  | | | |  | |  |
|  | Chemotherapy | 1785 | 1 |  |  | 1 | | | |  | |  |
|  | None | 1172 | 0.50 | 0.42 – 0.59 | <0.01 | 0.44 | | | | 0.35 – 0.55 | | <0.01 |
|  | Other neoadjuvant therapy | 74 | 1.19 | 0.67 – 2.28 | 0.58 | 1.10 | | | | 0.57 – 2.10 | | 0.78 |
| Surgical procedure |  |  |  |  |  |  | | | |  | |  |
|  | Minimally invasive | 1431 | 1 |  |  | 1 | | | |  | |  |
|  | Open | 1602 | 0.78 | 0.66 – 0.92 | <0.01 | 0.93 | | | | 0.75 – 1.14 | | 0.47 |
| Hospital volume (gastric resections per year) |  |  |  |  |  |  | | | |  | |  |
|  | < 20 | 856 | 1 |  |  | 1 | | | |  | |  |
|  | 20 – 39 | 1786 | 1.17 | 0.98 – 1.41 | 0.09 | 1.07 | | | | 0.84 – 1.36 | | 0.61 |
|  | ≥ 40 | 392 | 1.41 | 1.06 – 1.87 | 0.02 | 1.32 | | | | 0.93 – 1.87 | | 0.12 |
| Year of resection |  |  |  |  |  |  | | | |  | |  |
|  | < 2016 | 1626 | 1 |  |  | 1 | | | |  | |  |
|  | 2016 and later | 1406 | 1.26 | 1.07 – 1.49 | <0.01 | 1.19 | | | | 0.97 – 1.46 | | 0.10 |

Legend:

*Online Supplements Table 2. Univariable and multilevel multivariable logistic regression model, nested for factorized hospital identification number, to assess the association of patient, tumor, hospital, and treatment characteristics with curative surgery (no failure to cure) for gastric cancer stage II or higher.*

A American Society of Anesthesiologists Score

B Charlson Comorbidity Index

C In conformity with the 7th edition of the TNM rules for classification

D Adjusted Odds Ratio


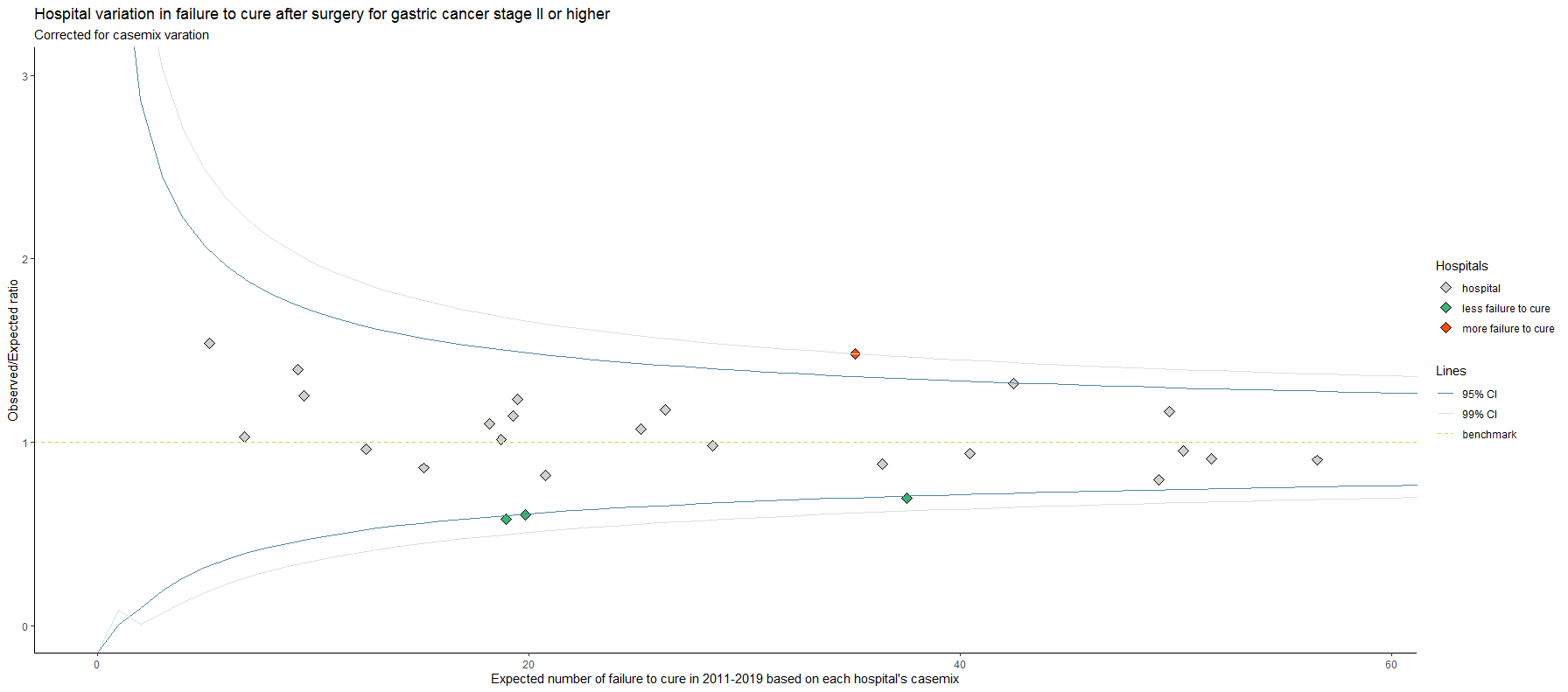


*Online Supplements Figure 1. Supplemental Digital Content Figure 1. Case-mix corrected funnel plot showing significant hospital variation in failure to cure after gastric cancer surgery stage II or higher.*
